# Yescarta: A New Era for Non-Hodgkin Lymphoma Patients

**DOI:** 10.7759/cureus.11504

**Published:** 2020-11-16

**Authors:** Salma M AlDallal

**Affiliations:** 1 Hematology, Amiri Hospital, Kuwait City, KWT

**Keywords:** axicabtagene ciloleucel, cd19, yescarta, non-hodgkin lymphoma, car t-cell, lymphoma

## Abstract

The use of conventional therapeutic approaches in patients with lymphoma demonstrates significant drug resistance leading to poor prognosis with reduced median survival period. T-cell immunotherapy has diverted huge attention of the researchers in recent times to engage in the stated research studies in the pool of chemotherapy-refractory lymphoma patients. B-cell antigen CD19-targeted chimeric antigen receptor (CAR) T-cell products are approved for the treatment of non-Hodgkin B-cell refracting or relapsing lymphoma. The aim of this article is to give an idea about the use of FDA-approved anti-cancer gene therapy, Axicabtagene ciloleucel, marketed under the name of Yescarta®. Axicabtagene ciloleucel is developed from the patients’ mononuclear peripheral blood cells during which T cells are orchestrated to articulate a CAR that diverts them to identify CD19-expressing cells. It is used in patients with non-Hodgkin B-cell refracting or relapsing lymphoma who had no response to prior therapeutic regiment involving the use of chemotherapeutics. Here, we review the mode of action, safety, and efficacy of Yescarta.

## Introduction and background

In the United States, lymphoma is known to be the most prevalent hematological malignancy, which accounts for 3.5% of all cancer-related death. Depending on the cell origin, they can be narrowly categorized as B-cell, T-cell, or natural killer/T-cell lymphomas. The most common form of lymphoma, B-cell lymphoma, can be further divided into Hodgkin lymphoma and non-Hodgkin lymphoma (NHL). NHL, accounting for approximately 90% of cases of B-cell lymphoma, is differentiated into forms such as diffuse large B-cell lymphoma (DLBCL) or follicular lymphoma [1].

Combinatorial therapies involving radiotherapy, immunotherapy/chemotherapy, and stem cell hematopoietic transplantation are the conventional therapies for the treatment of lymphoma. However, drug resistance to conventional therapies develops in approximately 20% of all lymphoma patients, resulting in a decrease in therapeutic efficacy [2,3]. In such a scenario, new therapies need to be designed, which can boost therapeutic outcomes in patients with recurrent or treatment-refractory lymphoma.

T cells have surfaced as a major contributor in the immune response for the amelioration of cancer. T cells are genetically modified to tumor targets to express a chimeric antigen receptor (CAR), which presents immense potential as a therapeutic option in the treatment of cancer [4]. The results of initial stage clinical trials have reported significant efficacy of targeted CD19-specific CAR T cells in treating chemoresistant B-cell malignancies [5]. Till date, two genetically modified T-cell immunotherapy products have been approved by the FDA. The first one being tisagenlecleucel (Kymriah, Novartis, East Hanover, NJ, USA) for acute lymphoblastic leukemia of B cells and the second one is axicabtagene ciloleucel (axi-cel; Yescarta®, Kite Pharma, Santa Monica, CA, USA) for relapsed or refractory large B-cell lymphoma, including DLBCL [6]. The purpose of this review is to outline the mode of action as well as the safety and efficacy of Yescarta.

## Review

Yescarta

Yescarta was the second anti-cancer gene therapy approved in the year 2017 by the FDA. This gene therapy is focused on genetically altering a line of immune cells of a patient to make them effective against the tumor cells. The manufacturing of the novel drug, axi-cel, is done by Kite Pharma, and it is marketed as Yescarta. It was approved for the treatment of NHL patients who had not previously responded to conventional chemotherapy [7].

Mode of action

Axi-cel binds to normal B cells and CD19-expressing cancer cells. It targets downstream signaling cascades in CD19-expressing cancer cells. The engagement of CAR T-cell and CD19-expressing target cells leads to the activation of T-cell proliferation, leading to inflammatory cytokines release. This series of events results in CD19-expressing cell apoptosis [8]. Following the standard procedure on leukapheresis, peripheral blood cells of the patient are collected. The collected cells are expanded, harvested, and then formulated as a suspension. The cell suspension is infused in the patients. Upon reinfusion, anti-CD19 CAR T cells detect and eliminate target cells, which express CD19 [8].

ZUMA-1 clinical trial

The FDA approved the use of axi-cel for the treatment of patients with relapsed or refractory aggressive B-cell NHL based on the appreciable results on the safety and efficacy from the ZUMA-1 clinical trial [8].

Although axi-cel is manufactured in a central facility, the patients are usually pre-conditioned with a chemotherapeutic regimen for a three-day duration prior to the start of the drug for enhancing the CAR T-cell infusion. Kite Pharma upon collaboration with the National Cancer Institute has evaluated lower doses of conditioning therapy. The conditioning therapy comprises cyclophosphamide and fludarabine administered systemically at a dose of 300-500 mg/m^2^ and 30 mg/m^2^, respectively, daily for a time span of three days [9]. This pre-conditioning therapy resulted in effective therapeutic efficacy against advanced lymphomas as compared to the response rates involving a high dose of pre-conditioning. Although the specific mechanism related to the use of specified chemotherapeutics for the enhancement of the therapeutic efficiency is still under rigorous investigation, it has been reported that a rise in the level of systemic interleukin (IL)-15 and chemokines is observed upon cyclophosphamide and fludarabine conditioning. This results in improving the efficacy of CAR T cell activity [10]. Details of the ZUMA-1 clinical trial are presented in Table 1.

**Table 1:** Details of the ZUMA-1 clinical trial CAR, chimeric antigen receptor; CLL, chronic lymphocytic leukemia; CR, complete response rate; CRS, cytokine release syndrome; DLBCL, diffuse large B-cell lymphoma; HLH, hemophagocytic lymphohistiocytosis; ORR, objective response rate; PFS, progression-free survival; PMBCL, primary mediastinal B-cell lymphoma; TFL, transformed follicular lymphoma

	Details
Study design	Single-arm, open-label
Clinical trial phase	Phase 2
Patients (median age)	101 (58 years)
Inclusion criteria	Patients with DLBCL, TFL, and PMBCL were included in the study.
Exclusion criteria	Patients with Richter transformation from CLL, indolent lymphoma, and mantle cell lymphoma were excluded from the study.
Dose	2 × 10^6^ CAR-positive viable T cells/kg of body weight as a single intravenous infusion; maximum permitted dose: 2 × 10^8^ CAR-positive viable T cells/kg of body weight.
Efficacy	The median time to response was observed to be 1 month (range: 0.8–6 months). ORR was observed in 82% of patients enrolled. CR was observed in 54% of patients enrolled. Six-month PFS was noted in 49% of enrolled patients. Incidences of relapses appeared to be infrequent after a time period of 6 months.
Toxicity	CRS grade 3 or higher in 13% of the enrolled patients. Neurologic events grade 3 or higher in 28% of the enrolled patients.
Death incidences	Progression of disease: 37 patients. CRS-associated events such as cardiac arrest, HLH: 2 patients, pulmonary embolus: 1 patient.

Manufacturing

The optimum manufacturing processes of the production of CAR T cells comprise harvesting T cells and selection of T cells with the use of magnetic beads. In the presence of IL-2 and an anti-CD3 antibody, T-cell activation occurs. This is followed by the CAR gene introduction into the activated T cells through the inclusion of a vector, leading to the expansion of CAR T cells and formulation of products involving CAR T cells [11]. With an aim to streamline the procedure and boost its use for broad global trials, several primary measures have been undertaken to manufacture axi-cel, with the major ones being making the culture media by eliminating human serum. Hence, the risk related to incidence of infection is decreased and the utilization of magnetic beads for the selection of T cells is also avoided. Additionally, there seemed to be a reduction in the period of ex vivo growth, thereby reducing the possibility of exhaustion of T cells [12]. This procedure contributed to the development of primarily CD3+, CD4+ with CD8+ T cells, with the remainder being effector T cells and central memory T cells. The procedure was strongly efficient, and the predicted time period for axi-cel production was approximately two weeks [13].

Adverse effects

The far more acute toxicity, which develops within hours to a few days following Yescarta infusion, mainly comprises cytokine release syndrome (CRS) and neurological events (NEs). The precipitation of the aforementioned adverse effects is normally reversible, which typically occurs within two weeks without enduring complications in the majority of the patients [14].

Within 12 hours of infusion, the clinical features of CRS may be clearly apparent. The initial symptoms include fever and certain constitutional symptoms. The other noted adverse effects include hypoxemia, hemodynamic instability, and damage to organs such as the heart and kidney. CRS is believed to have originated from pervasive and synchronized T-cell activation. The activation causes a release of soluble inflammatory mediators, which includes cytokines, chemokines, and immune-effectors [15]. CRS is correlated with high levels of interferon (IFN)-γ, GM-CSF (granulocyte-macrophage colony-stimulating factor), IL-6, IL-10, ferritin, and C-reactive protein [16]. Higher grades of NEs are associated with an elevated level of IL-15, granzyme B, and IL-10. Tachycardia, fatigue, headache, decreased appetite, chills, diarrhea, and febrile neutropenia are some of the noted adverse events associated with the administration of Yescarta [17].

## Conclusions

With the rapid development pertaining to research in CAR T cells, more number of products in clinical usage is foreseen in a couple of years. Future efforts need to be taken aimed at reducing the associated toxicity while retaining the therapeutic efficacy. Researchers should focus on a multitude of approaches such as the inclusion of functionality in CAR T cells, tuning the dosing strategies, and planning detailed studies correlating safety and efficacy. It is needful to revisit and evaluate rational CAR T-cell combinations with certain immunotherapies and novel cancer therapeutic regimens to showcase better clinical outcomes.

## References

[1] Howlader N, Noone AM, Krapcho M (2020). SEER Cancer Statistics Review (CSR) 1975-2014. https://seer.cancer.gov/archive/csr/1975_2014/.

[2] Arcaini L, Rossi D, Paulli M (2016). Splenic marginal zone lymphoma: from genetics to management. Blood.

[3] Kuruvilla J (2016). The role of autologous and allogeneic stem cell transplantation in the management of indolent B-cell lymphoma. Blood.

[4] Lim WA, June CH (2017). The Principles of Engineering Immune Cells to Treat Cancer. Cell.

[5] Sadelain M, Rivière I, Riddell S (2017). Therapeutic T cell engineering. Nature.

[6] Lulla PD, Hill LC, Ramos CA, Heslop HE (2018). The use of chimeric antigen receptor T cells in patients with non-Hodgkin lymphoma. Clin Adv Hematol Oncol.

[7] Viardot A, Wais V, Sala E, Koerper S (2019). Chimeric antigen receptor (CAR) T-cell therapy as a treatment option for patients with B-cell lymphomas: perspectives on the therapeutic potential of Axicabtagene ciloleucel. Cancer Manag Res.

[8] (2020). Yescarta. Santa Monica, CA: Kite Pharma; October.

[9] Kochenderfer JN, Dudley ME, Kassim SH (2015). Chemotherapy-refractory diffuse large B-cell lymphoma and indolent B-cell malignancies can be effectively treated with autologous T cells expressing an anti-CD19 chimeric antigen receptor. J Clin Oncol.

[10] Siddiqi T, Neelapu S, Locke F (2016). Updated phase 1 results from ZUMA- 1: a phase 1-2 multicenter study evaluating the safety and efficacy of KTE-C19 (anti- CD19 CAR T cells) in refractory aggressive B-cell non- Hodgkin lymphoma (NHL). Mol Ther.

[11] Roberts ZJ, Better M, Bot A, Roberts MR, Ribas A (2018). Axicabtagene ciloleucel, a first-in-class CAR T cell therapy for aggressive NHL. Leuk Lymphoma.

[12] Better M, Chiruvolu V, Oliver J (2016). Production of KTE-C19 (anti-CD19 CAR T cells) for ZUMA- 1: a phase 1/2 multi-center study evaluating safety and efficacy in subjects with refractory aggressive non-Hodgkin lymphoma (NHL). Mol Ther.

[13] Neelapu SS, Locke FL, Bartlett NL (2017). Axicabtagene Ciloleucel CAR T-cell therapy in refractory large B-cell lymphoma. N Engl J Med.

[14] Penfold T (2020). AACR 2017 | Primary results from the pivotal ZUMA-1 trial of KTE-C19 in refractory aggressive Non-Hodgkin Lymphoma patients. LymphomaHub.

[15] Lee DW, Gardner R, Porter DL (2014). Current concepts in the diagnosis and management of cytokine release syndrome. Blood.

[16] Maude SL, Barrett D, Teachey DT, Grupp SA (2014). Managing cytokine release syndrome associated with novel T cell-engaging therapies. Cancer J.

[17] Bot A, Rossi J, Yizhou J (2015). Cyclophosphamide and fludarabine conditioning chemotherapy induces a key homeostatic cytokine profile in patients prior to CAR T cell therapy. ASH Ann Meeting Abstracts.

